# Cheilitis Glandularis: Two Case Reports of Asian-Japanese Men and Literature Review of Japanese Cases

**DOI:** 10.5402/2011/457567

**Published:** 2010-12-15

**Authors:** Toru Yanagawa, Akira Yamaguchi, Hiroyuki Harada, Kenji Yamagata, Naomi Ishibashi, Masayuki Noguchi, Kojiro Onizawa, Hiroki Bukawa

**Affiliations:** ^1^Department of Oral and Maxillofacial Surgery, Clinical Sciences, Graduate School of Comprehensive Human Sciences, University of Tsukuba, 1-1-1 Tennodai, Tsukuba, Ibaraki, 305-8575, Japan; ^2^Section of Oral Pathology, Division of Oral Health Sciences, Department of Oral Restitution, Graduate School Medical and Dental Sciences, Tokyo Medical and Dental University, Tokyo 113-8549, Japan; ^3^Section of Oral and Maxillofacial Surgery, Division of Oral Health Sciences, Department of Oral Restitution, Graduate School Medical and Dental Sciences, Tokyo Medical and Dental University, Tokyo 113-8549, Japan; ^4^Department of Pathology, Life System Medical Sciences, Graduate School of Comprehensive Human Sciences, University of Tsukuba, 1-1-1 Tennodai, Tsukuba, Ibaraki 305-8575, Japan

## Abstract

Cheilitis glandularis (CG) is a rare disorder characterized by swelling of the lip with hyperplasia of the labial salivary glands. CG is most frequently encountered in the lower lip, in middle-aged to older Caucasian men; however Asian cases were rarely reported. In this paper we present two cases of CG in Asian-Japanese men. One was a 23-year-old male with CG of the superficial suppurative type. The other was a 54-year-old male with deep suppurative type. We also reviewed the Japanese cases of CG in the literature and discussed about clinical feature of Japanese CG.

## 1. Introduction

Cheilitis glandularis (CG) is a rare disorder characterized by swelling of the lip with hyperplasia of the labial salivary glands. The term cheilitis glandularis was first used by Volkmann in 1870 to describe a disorder that presented as a chronic, suppurative inflammation of the lower lip, characterized by swelling of the mucous glands and associated with mucopurulent discharge through dilated ductal openings [1, 2]. The histopathologic features of CG show nonspecific chronic inflammation of minor salivary gland tissue. Differential diagnosis of the lip edema may include angioedema, exfoliative cheilitis, cheilitis granulomatosa, or elephantiasis nostras [3].

Cheilitis glandularis is most frequently encountered in the lower lip, in middle-aged to older Caucasian men [4, 5]; Japanese cases are very rare. In this paper we present two cases of CG in Asian-Japanese men. One was a 23-year-old male with swelling of both lips and superficial ulceration. The diagnosis was CG of the superficial suppurative type. The other was a 54-year-old male with upper lip swelling and abscess formation. This case was determined to be the deep suppurative CG type and was treated by surgical excision. We also review the Japanese cases of CG in the literature and discuss the features of Asian-Japanese CG in Japan.

## 2. Case Reports

**Case 1**

**Figure 1:**
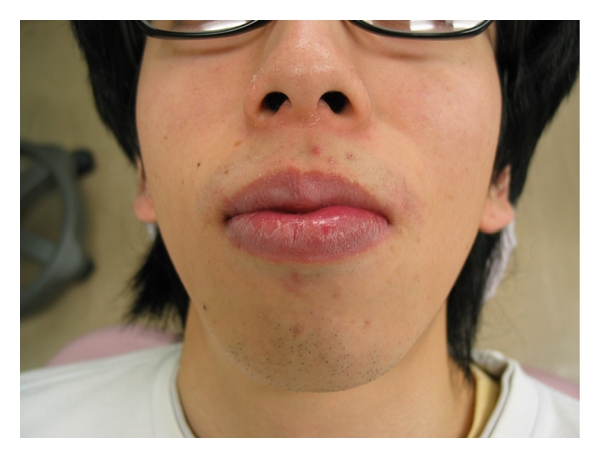
Extraoral photograph of the patient in Case 1 showing the everted lower lip.

**Figure 2:**
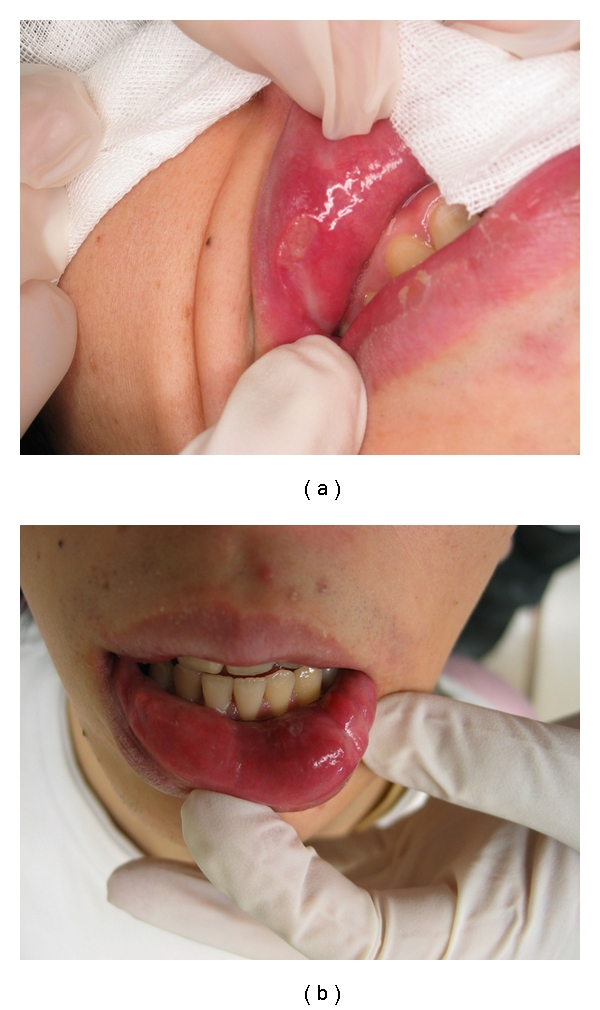
(a) and (b) show the upper and lower lip of the patient, respectively. There was painless swelling, and induration on both lips with abscess. The surface mucosa showed a color change and produced cloudy fluid at the sites of ductal openings.

**Figure 3:**
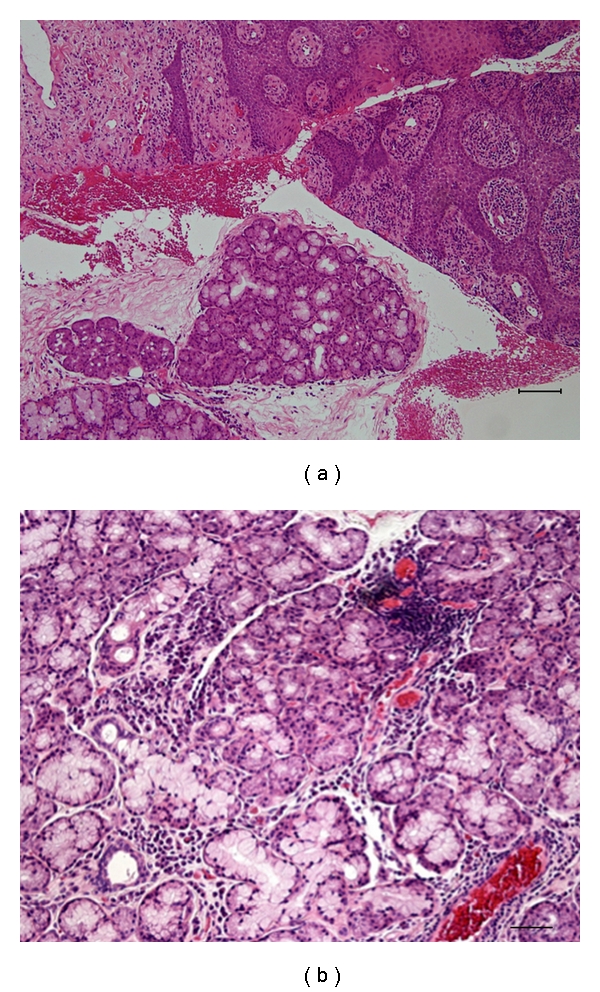
Histologic findings of Case 1. Focal infiltration of lymphocytes and plasma cells is observed at interlobular region in the slightly hypertrophic labial gland. Original magnification x40 (a), x200 (b).

A 23-year-old Asian-Japanese man consulted the Department of Oral and Maxillofacial Surgery of Tsukuba University Hospital complaining of upper and lower lip swelling with superficial ulceration in August 2005. He noticed these masses at first in August 2004 and visited dermatology medical practitioner. He had applied cepharanthine, hochuekkito (chinese herbal remedy), triamcinolone acetonide, irsogladine maleate, and koujin powder (chinese herbal remedy) as recommended by his dermatologist, and the swelling was reduced after two months of treatment. Four months later the swelling of both lips recurred following a small superficial ulceration, and it was reduced again two months later. In June 2005 the swelling of both lips recurred, but there was no resolution of the symptoms for more than 2 months. The patient consulted his dentist, who referred him to our department for further evaluation and treatment. His previous history revealed nothing remarkable. The patient had no known drug allergies but did have a food allergy to shellfish. Clinical examination revealed a well-developed and nourished man in no apparent distress. Intraoral examination revealed good dental health. Extraoral examination revealed that the left side of his lower lip was enlarged and grossly everted, and the right side of his upper lip was slightly everted and rather firm to palpation with indiscernible borders (Figure 1). The left lower and the right upper labial mucosa had a round ulceration, approximately 8 mm in diameter (Figures 2(a) and 2(b)), and produced cloudy exudate at ductal openings. A biopsy of the tumescent region including the mucosal area in the lower lip was carried out. Histologically, the epithelium was slightly thickened, and moderate inflammatory cell infiltration with lymphocytes, plasma cells, and neutrophiles was observed at subepithelial connective tissues. The labial glands were slightly hyperplastic and associated with focal infiltration of lymphocytes and plasma cells at interlobular region (Figure 3). These findings were compatible with CG. We informed the patient of the diagnosis, but because he did not wish to have the affected regions removed surgically, followup was continued. The enlargement of both lips resolved spontaneously within a month, and there has been no relapse in two years.

**Case 2**

**Figure 4:**
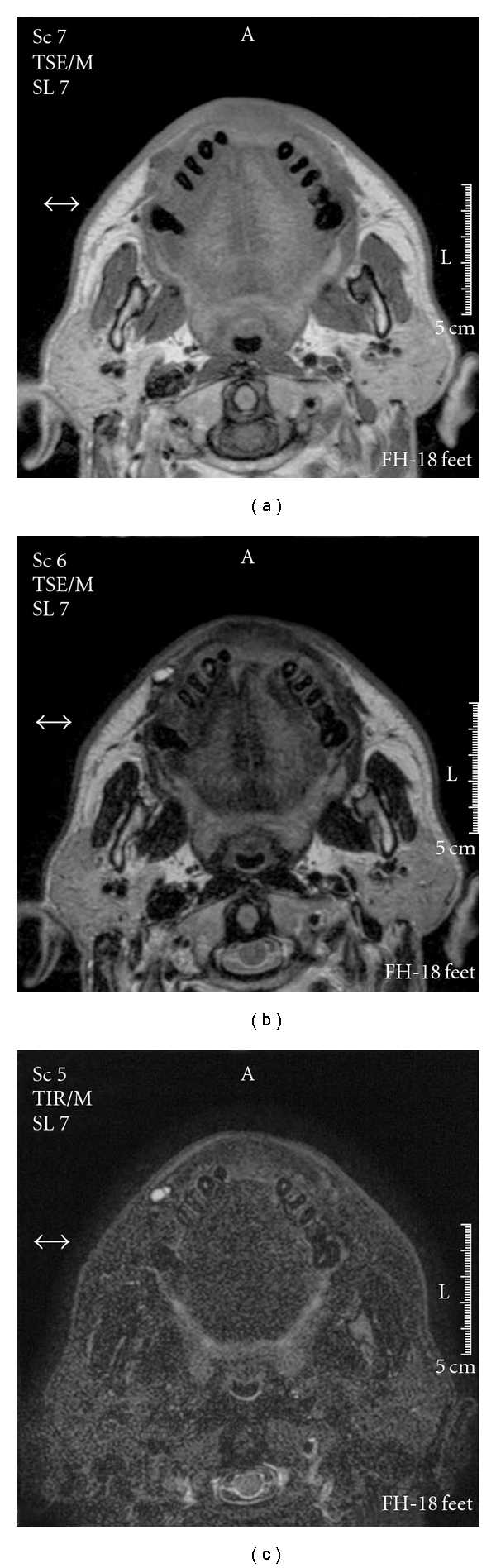
MR imaging of Case 2. (a) T1-weighted image, (b) T2-weighted image, and (c) short T1 inversion recovery image.

**Figure 5:**
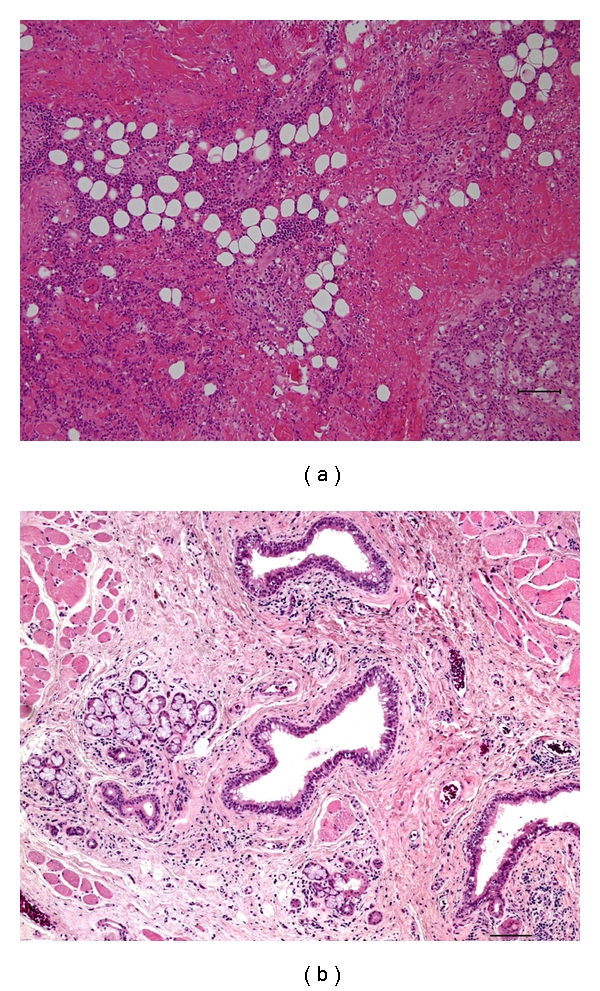
Histologic findings of Case 2. Incisional biopsy (a) and wedge excision of the upper lip (b). Atrophic labial glands and dilated ducts were scattered among fibrosis associating with lymphocyte infiltration. Original magnification x40 (a), (b).

A 54-year-old Asian-Japanese man consulted the Department of Oral and Maxillofacial Surgery of Tsukuba University Hospital complaining of upper lip swelling like phlegmonous abscess. He noticed small masses in his upper lip in October 2005 but had not sought treatment. In January 2006 these masses suddenly became inflamed, and he consulted a dentist, who referred him to Tsukuba University Hospital. The patient had a history of hypertension but was not on medication for it, and he had no known allergies. Clinical examination revealed a well-developed and nourished man in no apparent distress. Extraoral examination revealed a lesion that was firm to palpation with indiscernible borders, and diffuse swelling measuring approximately 3.0 × 2.0 cm and extending superiorly from the right upper lip to the cheek region and posteriorly to the angle of the mouth. Intraoral examination revealed a firm, submucosal swelling in the right buccal mucosa, extending from the maxillary right first permanent molar to the lip region. There was a fistula in the center of the lesion in the mucous membranes of the inside right upper lip. There was severe periodontitis around the upper molar teeth, but probe examination indicated that the fistula was cecal and 20 mm deep, and no connection was found between the periodontal gingiva and the lip. Infectious focus is not clear, so we suspected cheilitis, and incisional biopsy of the nodular region including the mucosal area was carried out. Histologically, the labial glands were surrounded by thick fibrous connective tissues associating with lymphocyte infiltration and diagnosed as cheilitis. Fibrosis and lymphocyte infiltration also occurred at interlobular region in the labial gland (Figure 5(a)). In a week the inflammation was resolved by drainage and antibiotic medication, but a small nodule with a mucus-discharging punctum remained. A cystic lesion (9 × 4 mm) was detected in the upper lip by magnetic resonance imaging (MRI). The signal intensity of the mass was relatively low compared with the surrounding soft tissue on T1-weighted magnetic resonance (MR), but remarkably high on T2-weighted and short T1 inversion recovery MR, which suggested fluid collection (Figures 4(a), 4(b), and 4(c)). Five months later, the patient had a persistent nodule that was exudative in his upper lip, and he was scheduled for surgical excision of the nodule. We designed a spindle-shaped incisional line on the nodule that included the opening of the duct and excised the nodule along with the surrounding scar tissue under a general anesthetic. The tissue specimen submitted for histopathologic examination measured 1.5 × 1.0 × 1.0 cm. Histologically, extensive fibrosis associating with lymphocyte infiltration was observed at the central region of the specimen. A few atrophic labial glands and dilated duct were scattered among the fibrosis (Figure 5(b)). These findings were compatible with CG. The patient is currently being seen on a regular recall basis and is doing well after 1 year.

## 3. Discussion

Cheilitis glandularis (CG) is a rare disorder characterized by swelling of the lip with hyperplasia of the labial salivary glands. CG was classified into three types by Schauermann: simple, superficial suppurative, and deep suppurative [6, 7], and this classification is commonly used. The simple type consists of multiple painless lesions that exhibit openings and dilated ducts. It lacks inflammation, but mucinous material is extruded when the lip is squeezed, and numerous small nodules can be felt on palpation. If these lesions are infected, the disease may progress to the superficial or deep suppurative type. Superficial suppurative type GC is thought to result from a secondary infection of the simple type. This GC type is characterized by painless crusting, swelling, and induration of the lip with superficial ulceration. The surface mucosa shows a color change and produces clear to cloudy fluid at the sites of ductal openings. Deep suppurative type GC is a deep-seated infection accompanied by abscess formation and fistulous tracts and is thought to be associated with chronic infection [8]. Many different therapeutic modalities have been reported for GC, including antibiotics, radiotherapy, and steroids, but surgery, that is, vermilionectomy or lip strip, is recommended. Recurrence after surgery is rare [3, 7, 9]. 

Cheilitis glandularis is most frequently encountered in the lower lip, in middle-aged to older Caucasian men [4]; however, our cases were in Asian-Japanese men. In the first case, both lips were affected, and superficial suppurative type CG was diagnosed. In the second, the upper lip was affected and deep suppurative type CG was diagnosed. Asian-Japanese cases of CG are very rare and are mainly reported in Japanese domestic journals. To our knowledge, 17 cases, including ours, have been reported in the domestic literature between 1952 and 2009 [7, 10, 11]. Our findings from this review of Japanese cases are summarized in Table 1. The mean age is 58 years (range 1 to 76) and the sex ratio is 6 : 11 (f : m). In this literature search, 11 of the 17 Japanese cases affected the upper lip or included both lips. There are only two reported cases of the simple type. Among the reported cases of GC in the international literature, few involve the upper lip [12]. However, our literature search showed that the upper lip of Asian-Japanese patients was often affected. The reason for this difference in morbidity is not clear, but it may reflect racial or etiological differences. The etiology of CG is still unknown, but it has been suggested to be an autosomal dominant hereditary disease for which smoking, poor oral hygiene, chronic exposure to sunlight and wind, and a compromised immune system are predisposing factors [2, 8, 9, 13, 14]. Swerlick and Cooper reviewed forty-eight cases of CG described in the literature and suggested that CG patients can be categorized into three groups [15]. The largest group develops CG as a result of marked, chronic sun and wind exposure. The second group has a history suggesting a coexistent atopic diathesis. The third group has factitious cheilitis. In their review, CG represents the usual clinical presentation of cheilitis that develops after chronic exposure to one or more irritants. Our presented cases did not have obvious causes of CG; however, in the first case, diathesis is suspected because the patient had a food allergy. In the second case, the patient had periodontitis of a molar tooth near the CG, which could act as a chronic irritant to facilitate CG development. 

CG is an unusual disease, characterized by a painless, enlarged everted lip with mucoid-draining pores, that may be encountered in clinical practice. In some cases, CG may degenerate into squamous cell carcinoma [5, 16, 17]. However, like our first case, it is sometimes self-healing. Thus, there may be many unreported cases of CG in which the patient does not seek treatment because the CG resolves spontaneously. Further research of CG, including thorough investigation of its etiology, is necessary to improve our understanding of this disease.

## 4. Conclusion

We reported two cases of CG affecting the upper and both lips in Asian-Japanese men. One was superficial suppurative type and the other was deep suppurative type. 

## Figures and Tables

**Table 1:** Clinical feature of 15 cases of cheilitis glandularis in Japan from the literature.

Year	Author	Location	Type	Age	Sex
1952	Tsukada et al.	Lower lip	Simple	41	Female
1974	Saito et al.	Lower lip	Simple	62	Female
1976	Kuroi et al.	Both lips	Superficial suppurative	1	Female
1979	Nagayama et al. [10]	Both lips	Superficial suppurative	53	Male
1983	Miyazaki et al. [11]	Upper lip	Superficial suppurative	64	Male
1983	Miyazaki et al. [11]	Upper lip	Deep suppurative	70	Male
1985	Shimizu et al.	Both lips	Superficial suppurative	76	Male
1986	Koh et al.	Upper lip	Superficial suppurative	63	Male
1989	Matsumoto et al. [7]	Upper lip	Superficial suppurative	63	Male
1990	Takenoshita et al.	Lower lip	N.D.	64	Male
1994	Ioroi et al.	Upper lip	Deep suppurative	72	Female
2000	Ishioka et al.	Upper lip	N.D.	67	Female
2002	Ohtake et al.	Lower lip	N.D.	58	Male
2008	Ninomiya et al.	Lower lip	N.D.	73	Female
2008	Ninomiya et al.	Buccal mucosa	N.D.	69	Male
2009	Yanagawa et al.	Both lips	Superficial suppurative	23	Male
2009	Yanagawa et al.	Upper lip	Deep suppurative	54	Male

N.D.: not determined

## References

[1] von Volkmann R (1870). Einige Fälle von Cheilitis glandularis apostematosa (Myxadenitis labialis). *Archiv für Pathologische Anatomie und Physiologie und für Klinische Medicin*.

[2] Lederman DA (1994). Suppurative stomatitis glandularis. *Oral Surgery, Oral Medicine, Oral Pathology*.

[3] Stoopler ET, Carrasco L, Stanton DC, Pringle G, Sollecito TP (2003). Cheilitis glandularis: an unusual histopathologic presentation. *Oral Surgery, Oral Medicine, Oral Pathology, Oral Radiology, and Endodontics*.

[4] Bender MM, Rubenstein M, Rosen T (2005). Cheilitis glandularis in an African-American woman: response to antibiotic therapy. *Skinmed.*.

[5] Nico MMS, Nakano de Melo J, Lourenço SV (2010). Cheilitis glandularis: a clinicopathological study in 22 patients. *Journal of the American Academy of Dermatology*.

[6] Schauermann H (1966). *Krankheiten der Mundschleimhaut und der Lippen*.

[7] Matsumoto H, Kurachi Y, Nagumo M (1989). Cheilitis glandularis: report of a case affecting the upper lip. *Showa Shigakkai Zasshi*.

[8] Weir TW, Johnson WC (1971). Cheilitis glandularis. *Archives of Dermatology*.

[9] Musa NJ, Suresh L, Hatton M, Tapia JL, Aguirre A, Radfar L (2005). Multiple suppurative cystic lesions of the lips and buccal mucosa: a case of suppurative stomatitis glandularis. *Oral Surgery, Oral Medicine, Oral Pathology, Oral Radiology and Endodontology*.

[10] Nagayama M, Nakata S, Oka T, Komoli A (1979). Cheilitis Glandularis: Report of a case affecting the upper lip. *Japanese Journal of Oral and Maxillofacial Surgery*.

[11] Miyazaki H, Takenoshita Y, Shinohara M, Oka M (1983). Cheilitis Glandularis purulenta in the upper lip: Report of two cases. *Japanese Journal of Oral and Maxillofacial Surgery*.

[12] Winchester L, Scully C, Prime SS, Eveson JW (1986). Cheilitis glandularis: a case affecting the upper lip. *Oral Surgery Oral Medicine and Oral Pathology*.

[13] Rada DC, Koranda FC, Katz FS (1985). Residents’ corner: cheilitis glandularis—a disorder of ductal ectasia. *Journal of Dermatologic Surgery and Oncology*.

[14] Yacobi R, Brown DA (1989). Cheilitis glandularis: a pediatric case report. *The Journal of the American Dental Association*.

[15] Swerlick RA, Cooper PH (1984). Cheilitis glandularis: a re-evaluation. *Journal of the American Academy of Dermatology*.

[16] Michalowski R (1962). Cheilitis glandularis, heterotopic salivary glands and squamous cell carcinoma of the lip. *The British Journal of Dermatology*.

[17] Reiter S, Vered M, Yarom, N, Goldsmith C, Gorsky M Cheilitis glandularis: clinico-histopathological diagnostic criteria.

